# Relationship Between Insulin Resistance Indicators and Type 2 Diabetes Mellitus in Romania

**DOI:** 10.3390/ijms26209888

**Published:** 2025-10-11

**Authors:** Adela-Gabriela Ştefan, Diana Clenciu, Ionela-Mihaela Vladu, Adina Mitrea, Diana-Cristina Protasiewicz-Timofticiuc, Maria-Magdalena Roşu, Theodora-Claudia Gheonea, Beatrice-Elena Vladu, Ion-Cristian Efrem, Delia-Viola Reurean Pintilei, Eugen Moţa, Maria Moţa

**Affiliations:** 1 Department of Diabetes, Nutrition and Metabolic Diseases, Calafat Municipal Hospital, 205200 Calafat, Romania; adela.firanescu@yahoo.com; 2 Department of Diabetes, Nutrition and Metabolic Diseases, Faculty of Medicine, University of Medicine and Pharmacy of Craiova, 200349 Craiova, Romania; diana.clenciu@umfcv.ro (D.C.); ionela.vladu@umfcv.ro (I.-M.V.); theodora.gheonea@umfcv.ro (T.-C.G.); 3 Department of Diabetes, Nutrition and Metabolic Diseases, Faculty of Midwives and Nursing, University of Medicine and Pharmacy of Craiova, 200349 Craiova, Romania; diana_protasiewicz@yahoo.com (D.-C.P.-T.); maria.rosu@umfcv.ro (M.-M.R.); 4 Faculty of Medicine, University of Medicine and Pharmacy of Craiova, 200349 Craiova, Romania; beatricevladu75@gmail.com; 5 Department of Internal Medicine—Medical Semiology, Faculty of Dentistry, University of Medicine and Pharmacy of Craiova, 200349 Craiova, Romania; 6 Department of Medical-Surgical and Complementary Sciences, Faculty of Medicine and Biological Sciences, “Stefan cel Mare” University, 720229 Suceava, Romania; delia.pintilei@usm.ro; 7 Consultmed Medical Centre, Department of Diabetes, Nutrition and Metabolic Diseases, 700544 Iasi, Romania; 8 Doctoral School, University of Medicine and Pharmacy of Craiova, 200349 Craiova, Romania; eugenmota@yahoo.com (E.M.); mmota53@yahoo.com (M.M.)

**Keywords:** insulin resistance, biomarkers, type 2 diabetes mellitus, PREDATORR study

## Abstract

Diabetes mellitus (DM) is a complex chronic disease, with a prevalence that has reached alarming proportions in recent decades. In this study, we aimed to analyze the association of type 2 diabetes mellitus (T2DM) with certain insulin resistance (IR) indicators, according to the gender of the participants enrolled in the PREDATORR study. Biomarkers such as the triglyceride–glucose (TyG) index and its derivates, triglyceride-to-high-density lipoprotein cholesterol (TG/HDL-c), and metabolic score for insulin resistance (METS-IR), as well as recent indicators, like cholesterol, HDL, the glucose (CHG) index and its derivates, CHG–body mass index (CHG-BMI), and CHG–waist circumference (CHG-WC), as well as its newly proposed derivates, such as CHG–waist-to-height ratio (CHG-WHtR), CHG–neck circumference (CHG-NC), and CHG–neck-to-height ratio (NHtRs were analyzed in 2080 subjects, divided into two groups, according to gender). Univariate and multivariate logistic regression was used to identify the relationships between IR indicators and T2DM. Regardless of gender, all the analyzed indicators presented statistically significantly higher values in T2DM (+) compared to T2DM (−). For both studied groups, CHG–WHtR had the largest AUROC curve: in males, the AUROC curve was 0.809, the cut-off value being 3.22, with a 70.7% sensitivity and 75.3% specificity; in females, the AUROC curve was 0.840, the cut-off value was 3.20, with a 79.3% sensitivity and 75.5% specificity, respectively. Regardless of gender, the age-adjusted model for multivariate logistic regression analysis demonstrated that TyG and CHG were predictive factors for T2DM.

## 1. Introduction

Diabetes mellitus (DM) is a complex chronic disease that requires ongoing medical care and is a major public health burden and a leading cause of death worldwide [1,2]. The prevalence of DM has reached pandemic proportions and continues to increase, registering alarming rates in recent decades in most developed and developing countries, due to increasing living standards and lifestyle changes [2,3]. In 2024, the International Diabetes Federation (IDF) estimated that there were 588.7 million people with DM globally, with a prevalence of 11.1%. The prevalence is expected to increase to 13% (852.5 million) by 2050, but predictions have often been exceeded [2]. The prevalence of DM in Romania was 11.6%, according to the PREvalence of DiAbeTes mellitus, prediabetes, overweight, Obesity, dyslipidemia, hyperuricemia and chRonic kidney disease in Romania (PREDATORR) study, conducted between 2012 and 2014. The prevalence of DM increased with age and was higher in men than in women [4].

Type 2 DM (T2DM) accounts for 90–95% of DM cases worldwide, and the main pathological mechanism involved in its development is insulin resistance (IR) [5,6]. Hyperglycemia usually develops gradually, and the early stages are often asymptomatic, so T2DM remains undiagnosed for several years. The classic symptoms of DM are caused by hyperglycemia and include polyuria, polydipsia, or unintentional weight loss. Moreover, even undiagnosed individuals are at an increased risk of developing a series of micro- and macrovascular complications, which can be difficult to manage due to the increased number of hospitalizations and even deaths [5,7].

IR usually precedes the diagnosis of T2DM by at least 10 years [8,9,10,11], with the decreased insulin sensitivity being associated with hyperglycemia, hyperuricemia, dyslipidemia, hypertension, endothelial dysfunction, and increased inflammatory markers, as well as the prothrombotic state, all of which lead to metabolic syndrome, T2DM, and metabolic dysfunction-associated steatotic liver disease (MASLD) [12,13,14]. IR assessment can thus identify individuals at an increased risk of developing T2DM and, furthermore, can improve the prediction and control of the disease progression [15,16]. Although the gold standard of IR assessment is represented by the technique developed by DeFronzo in 1979, namely the hyperinsulinemic-euglycemic clamp [17], it is complex, expensive, time-consuming, and difficult to apply for screening in clinical practice. The homeostasis model assessment of the insulin resistance (HOMA-IR) index has been used as an alternative [18]; it is an indirect method, but its applicability is limited, being expensive, and, on the other hand, measuring the serum insulin concentration is not a routine method and is susceptible to variations [19]. Given the limitations of the two methods, it is necessary to use simple and cost-effective tools for the assessment of IR.

Therefore, simple biomarkers that correlate with IR can identify individuals at an increased risk of developing T2DM, such as the triglyceride–glucose (TyG) index, its derivatives (TyG–body mass index (TyG–BMI), TyG–waist circumference (TyG–WC), TyG–waist-to-height ratio (TyG–WHtR)), triglyceride to high-density-lipoprotein cholesterol index (TG/HDL-c), and metabolic score for insulin resistance (METS-IR), but also recently developed indicators, which have proven their usefulness in the diagnosis of DM, such as cholesterol, HDL, the glucose (CHG) index and its derivatives, CHG–body mass index (CHG–BMI), and CHG–waist circumference (CHG–WC). These tools have been studied in patients with T2DM [8,12,20,21,22,23,24,25,26,27,28]. In the present study, we aimed to analyze these, as well as other recently proposed biomarkers of IR: TyG–neck circumference (TyG–NC) and TyG–neck-circumference-to-height ratio (TyG–NHtR) [29,30]. Similarly to these derivatives of TyG, we developed three new derivatives of CHG, namely, CHG–waist-to-height ratio (CHG–WHtR), CHG–neck circumference (CHG–NC), and CHG–neck-circumference-to-height ratio (CHG–NHtR). The present analysis was conducted within the framework of the PREDATORR study, which was a nationally representative cross-sectional survey conducted between 2012 and 2014 that enrolled 2728 Romanian adults aged 20–79 years to evaluate the prevalence of diabetes, prediabetes, obesity, dyslipidemia, hyperuricemia, and chronic kidney disease, thereby providing a comprehensive picture of metabolic health in Romania. While this survey generated essential epidemiological data, no information is available regarding the correlation between T2DM and IR indicators in this population. In this context, we aimed to investigate the relationship between multiple IR biomarkers and T2DM in a nationally representative Romanian cohort, with a particular focus on recently proposed CHG indices and three new derivatives (CHG–WHtR, CHG–NC, and CHG–NHtR). This approach allows us to extend previous research by introducing novel CHG-based markers and by comparing their performance with HOMA-IR, the most widely used surrogate of insulin resistance in epidemiological studies.

## 2. Results

In the PREDATORR study, 2728 subjects were enrolled, but the present study excluded patients diagnosed with prediabetes (580 subjects), as well as other forms of DM (39 cases), the majority of whom were represented by type 1 DM (36 subjects); moreover, data were incomplete for another 29 participants, so they could not be included in this analysis. Therefore, a total of 2080 subjects were considered eligible for the performed statistical analysis, most of them being women (52.3%). The prevalence of DM, as mentioned, was reported in a previously published article by colleagues from our team [4]. The characteristics of the study participants in terms of demographic, anthropometric, and paraclinical data are presented in Table 1.

In both groups, male and female, the analysis of demographic and anthropometric characteristics revealed statistically significant differences between T2DM (+) and T2DM (−) subjects, as follows: those with T2DM presented an older age, as well as significantly increased values of BMI, WC, WHtR, NC, NHtR, and SBP. In addition, paraclinical data demonstrated significantly increased values in participants with T2DM for both genders: FPG, HbA1c, HDL-c, and TG but also HOMA-IR. TC and LDL-c had significantly lower values in males with T2DM, but in females, the values were similar and thus, no statistical significance was recorded (*p* = 0.368 and 0.196, respectively).

Regarding lifestyle, a higher percentage of former smokers was found in the group of subjects T2DM (+), the differences being statistically significant both in females and males. Alcohol consumption, however, was declared in a higher percentage of participants without T2DM, the differences being significant only in the case of males. Reduced sleep duration was recorded more frequently in the group of females T2DM (+), and in the case of males, the results were similar (*p* = 0.667).

In Table 2, we calculated and compared the IR biomarkers according to the male and female groups but also according to the presence or absence of T2DM. It was found that, regardless of gender, all the analyzed indicators presented statistically significantly higher values in the T2DM (+) group of participants compared to T2DM (−) (*p* < 0.001).

**Figure 1:**
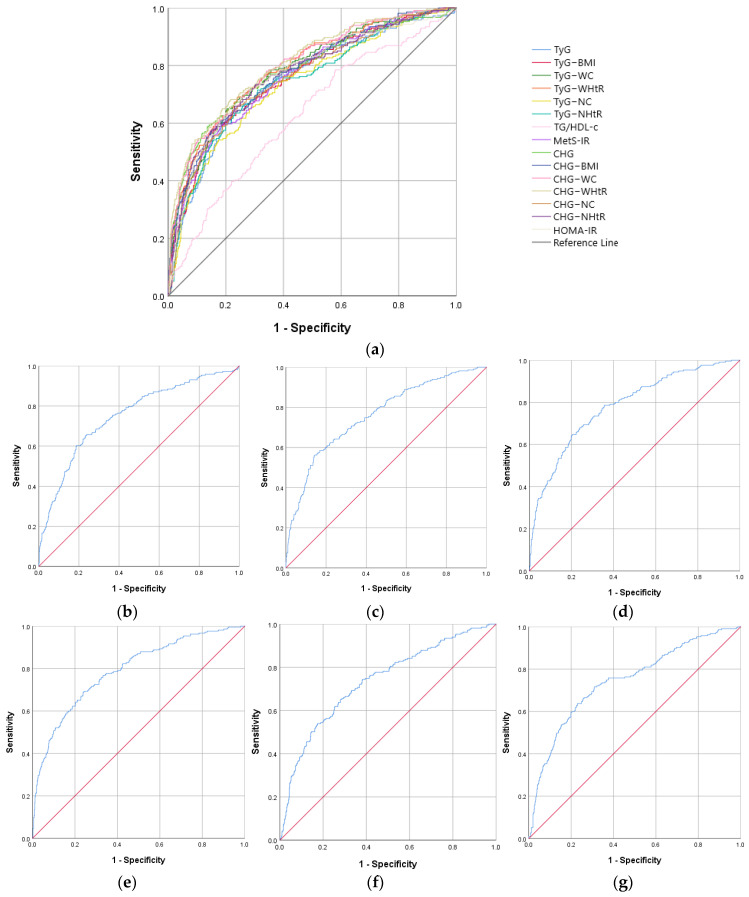

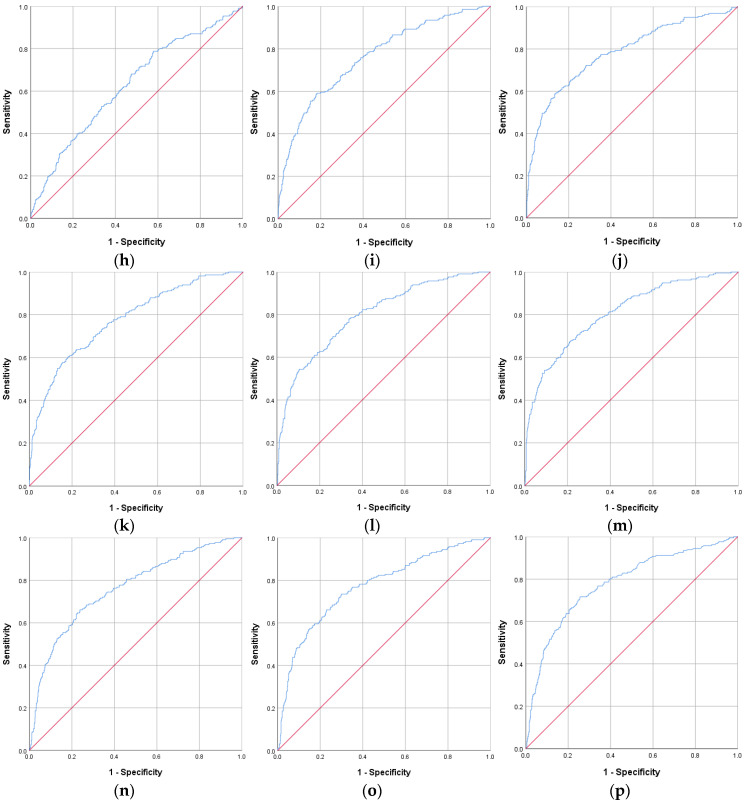
The ROC curve analysis for the IR biomarkers in males. (**a**) All IR indicators; (**b**) TyG; (**c**) TyG–BMI; (**d**) TyG–WC; (**e**) TyG–WHtR; (**f**) TyG–NC; (**g**) TyG–NHtR; (**h**) TG/HDL-c; (**i**) MetS-IR; (**j**) CHG; (**k**) CHG–BMI; (**l**) CHG–WC; (**m**) CHG–WHtR; (**n**) CHG–NC; (**o**) CHG–NHtR; and (**p**) HOMA-IR.

**Figure 2:**
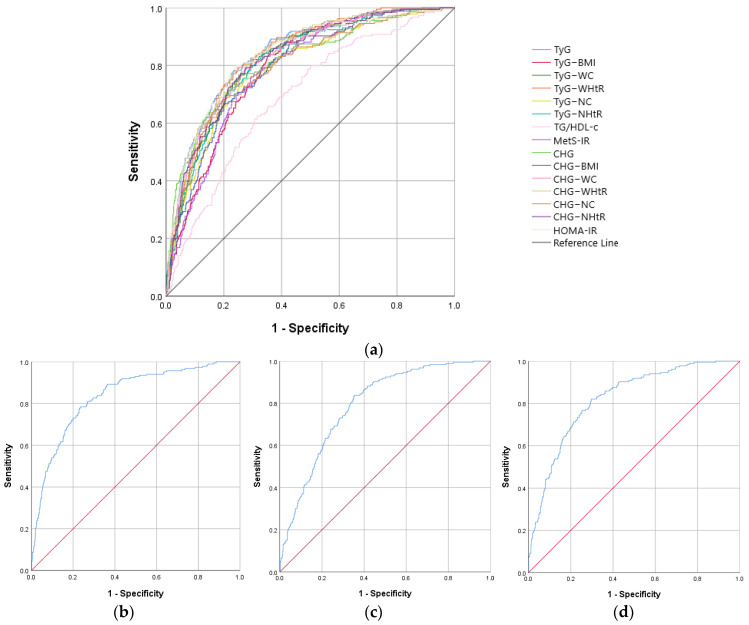

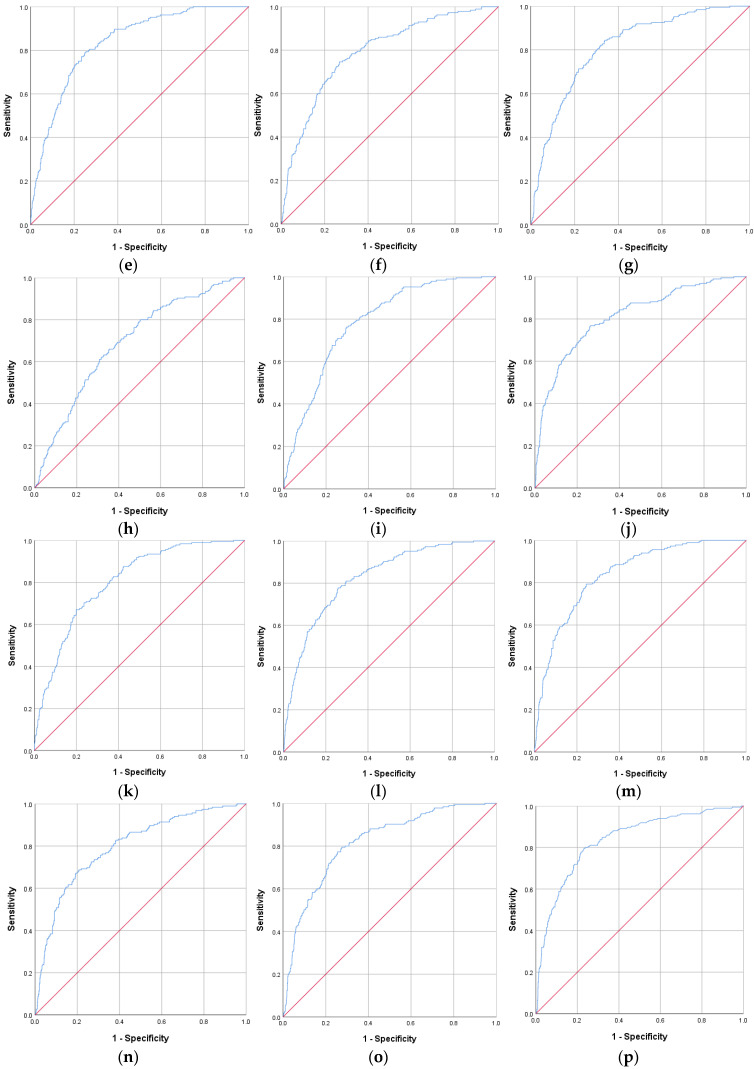
The ROC curve analysis for the IR biomarkers in females. (**a**) All IR indicators; (**b**) TyG; (**c**) TyG–BMI; (**d**) TyG–WC; (**e**) TyG–WHtR; (**f**) TyG–NC; (**g**) TyG–NHtR; (**h**) TG/HDL-c; (**i**) MetS-IR; (**j**) CHG; (**k**) CHG–BMI; (**l**) CHG–WC; (**m**) CHG–WHtR; (**n**) CHG–NC; (**o**) CHG–NHtR; and (**p**) HOMA-IR.

ROC curve analysis revealed, both in males and females, similar AUROC curves to HOMA-IR for TyG, TyG–WC, TyG–WHtR, CHG, CHG–WC, and CHG–WHtR, as well as a similar sensitivity and specificity. Data on all analyzed IR biomarkers are presented in Table 3 and Figure 1 for the male group, respectively, and Table 4 and Figure 2 for the female group.

In the male group, the largest AUROC curve of IR biomarkers was recorded in the case of CHG–WHtR with a value of 0.809, the cut-off value being 3.22, with a 70.7% sensitivity and 75.3% specificity (*p* < 0.001) (Table 3).

In the female’s group, the largest AUROC curve was also recorded in the case of CHG–WHtR with a value of 0.840, a cut-off value of 3.20, and, respectively, a 79.3% sensitivity and 75.5% specificity (*p* < 0.001) (Table 4).

The univariate logistic regression analysis revealed that almost all investigated variables were associated with T2DM in both groups. The exceptions were DBP, which did not reach statistical significance in either the male or female group, as well as TC and LDL-c, which were associated with T2DM only in the male group. The established limit for WHtR and NHtR represents the cut-off value of those parameters (Table 5).

Using an age-adjusted model for multivariate logistic regression analysis, we demonstrated that TyG and CHG were independent predictors of T2DM, regardless of gender. In addition, TyG–WC and CHG–NC were found to be independent predictors only in the male group. Moreover, the analysis showed that TG/HDL-c was less likely to be associated with T2DM both in males and females (*p* = 0.001 and *p* < 0.001, respectively). Regarding the other studied biomarkers, statistical significance was not reached (Table 6). In both univariate and multivariate logistic regressions, a limit was established for the analysis of TyG and its derivatives, TG/HDL-c, and MetS-IR, as well as for CHG and its derivatives, with this limit being represented by the cut-off value of each parameter, depending on gender; the values can be found in Table 4.

## 3. Discussion

In recent years, researchers have shown great interest in the relationship between certain indirect indicators of IR and T2DM in various population groups [24,27,31,32,33]. In the present study, we demonstrated their usefulness in diagnosing T2DM, with CHG–WHtR showing the highest AUROC values in both men and women, followed by CHG–WC and TyG–WHtR. In addition, TyG and CHG were independent predictors of T2DM in multivariate analysis, and some gender-related differences were observed in their diagnostic performance.

Consistent with the results obtained in the present study, Guo et al. found a positive correlation between TyG and the risk of developing T2DM, especially among female subjects [34]. Previously published studies have demonstrated the importance of TyG as a surrogate for IR, also demonstrating prediction for T2DM [35,36,37]. In a cohort study published in 2023, which included 5575 subjects, Xing et al. demonstrated the association between TyG and T2DM incidence, being also a more efficient predictor compared to its derivatives, namely TyG–BMI, TyG–WC, and TyG–WHtR; the predictive capacity recorded by TyG for the risk of T2DM is superior to other indicators in the case of female subjects, with the results being similar in our study [38]. Another study confirming these results was published by Zhang et al. describing a predictive effect of TyG on the risk of T2DM, which was higher in women compared to men [39].

The main mechanisms of T2DM development are represented by pancreatic β-cell dysfunction, as well as IR [16,40]. High blood glucose levels determine a proinflammatory status by increasing oxidative stress, causing an adverse environment for β-cells, subsequently to IR, and finally to β-cell mass and function loss [41,42]. Moreover, the increased level of free fatty acids (FFA), accompanied by the accumulation of TG in pancreatic β-cells promotes chronic β-cell dysfunction and damage [43]. Lipotoxicity negatively impacts glucose homeostasis, leading to glucotoxicity. Furthermore, lipo- and glucotoxicity induce cellular dysfunction, altering processes such as insulin gene transcription and insulin secretion, which underlie the development of T2DM [44]. These mechanisms highlight the importance of TyG as an independent predictor of T2DM, assessing both glucose and lipid metabolism [15].

A meta-analysis that included 32 studies proved the strong association between high TyG and T2DM development [45]; results are in accordance with the present study (TyG OR: 2.994 in males and, respectively, 8.170 in females). In a study published this year by colleagues from our team, it was demonstrated that TyG, as well as its derivatives, were associated with metabolic disorders, being significantly increased in subjects with T2DM [46]. Moreover, our team recently published data that highlighted the superior diagnostic value of TyG, being an independent predictive factor and demonstrating a superior ability to predict metabolic syndrome (MetS) compared to HOMA-IR [29]. Also, in a study published in 2022, our colleagues demonstrated the role of IR indicators as predictors of MASLD in patients with T2DM [47]. Although HOMA-IR is a supposedly superior method for assessing IR, as it reflects hyperglycemia and hyperinsulinemia, Yoon et al. demonstrated the better ability of TyG in predicting T2DM compared to HOMA-IR [48]; these results are not consistent with our study, which demonstrated a similar ability of the two indices, the discrepancy across countries already being suggested, but the explanation is probably represented by ethnic differences and anthropometric characteristics.

The present study also analyzed several derivatives of TyG (combinations with anthropometric indices), demonstrating a stronger association with prevalent T2DM in men, in the case of TyG–WHtR and TyG–WC (AUROC curve 0.791 and, respectively, 0.782) compared to HOMA-IR (AUROC curve 0.779) and even TyG (AUROC curve 0.751). Recent studies have proven the superior ability of TyG–BMI, TyG–WC, and TyG–WHtR in terms of predicting T2DM compared to TyG [24,33]. Contrary to our results, Xing et al. have highlighted the fact that TyG is a more efficient predictive factor compared to TyG–BMI, TyG–WC, and TyG–WHtR, as previously mentioned [38]. In our study, TyG-NC, as well as TyG-NHtR, had comparatively lower AUROC curves, but, nevertheless, they are useful biomarkers in the diagnosis of T2DM, with it being known that NC is an important indicator for MetS and cardiovascular risk [49,50]. Further research into these indicators is needed, as evidence is scarce.

In the present study, the TG/HDL-c index was a weak predictor for T2DM, the results being consistent with other research published in recent years, according to which TG/HDL-c presented a lower AUROC curve than TyG both in males and females [51,52].

The MetS-IR index, a biomarker developed by Bello-Chavolla et al., has proven, in recent studies, a predictive effect for identifying individuals at risk of developing T2DM, having a superior diagnostic capacity compared to TyG and TG/HDL-c [28,53,54]; the results are consistent with those obtained in our study in the male group. Other researchers demonstrated a significant association of high MetS-IR and the incidence of T2DM (OR: 1.804) [55], the results being in disagreement with our study, where the OR did not reach statistical significance, a fact that can be explained by differences regarding the study design.

In 2025, Mansoori et al. [12] published a study that investigated new biomarkers that can be used for T2DM diagnosis, namely, CHG, CHG–BMI, and CHG–WC. They were also compared with TyG and its corresponding derivatives, proving the superior diagnostic capacity of T2DM, as follows: CHG AUROC curve was 0.864 compared to 0.825 for TyG; CHG–BMI AUROC curve was 0.735 and, respectively, 0.698 for TyG–BMI; and AUROC curve of CHG–WC was 0.790 and 0.750 for TyG–WC. In accordance with these results, our study showed a stronger association with prevalent T2DM for CHG, CHG–BMI, and CHG–WC in the male group compared to TyG and its derivatives. Moreover, in the present study, along with TyG, CHG was significantly associated with the risk of T2DM both in the male (OR: 2.927, *p* < 0.001) and the female groups (OR: 2.004, *p* = 0.022). Among all the analyzed indicators, one of the three new biomarkers proposed by our team in this study, namely, CHG–WHtR, recorded the strongest discriminatory capacity for prevalent T2DM, even compared to HOMA-IR, in both of the studied groups: AUROC curves of 0.809 in men and, respectively, 0.840 in women. CHG–WHtR had a sensitivity and specificity superior to all other indicators (*p* < 0.001) but similar to HOMA-IR.

The strong discriminatory ability of CHG–WHtR, followed by CHG–WC and TyG–WHtR, suggests that these indices could be used to stratify individuals into risk categories for targeted screening and preventive interventions. Such markers may help identify persons who require further diagnostic evaluation, even in the absence of overt hyperglycemia. However, due to the cross-sectional design of our study, our results cannot be directly translated into a diagnostic algorithm. Prospective studies are needed to validate risk thresholds and to develop clinically applicable decision tools

These findings should be interpreted in the context of population-specific characteristics, taking into account that cut-off values for anthropometric and biochemical indicators are known to vary across ethnic groups and geographic regions. In this regard, the World Health Organization recommends lower BMI thresholds for Asian populations compared to European populations, reflecting the higher metabolic risk at lower levels of adiposity [56]. Likewise, WC and WHtR cut-offs have been shown to differ between European, Asian, and Latin American cohorts [57,58]. Therefore, while CHG–WHtR showed the strongest association with T2DM in our Romanian sample, these thresholds may not apply directly to other populations. External validation in cohorts with different ethnic, genetic, and lifestyle backgrounds is needed before the proposed cut-offs can be generalized.

However, this study has limitations that must be taken into account when interpreting the results. First, the study design is cross-sectional, which did not allow us to establish a cause–effect relationship between the analyzed factors at baseline and T2DM; changes in IR biomarkers over time could have provided additional data, as they could demonstrate a strong association with T2DM incidence. Second, the analysis was performed on a population from a specific geographical region, limiting the applicability of the results to populations with similar characteristics. In this situation, we must take into account differences in race or ethnicity, demographic factors, genetic background, access to public health services, environmental factors, and lifestyle patterns, as all of these may influence the assessment of T2DM risk. Third, our study compared IR biomarkers only with HOMA-IR, which, although widely used in epidemiological research, is itself an indirect surrogate of insulin resistance and not a true gold standard such as the hyperinsulinemic-euglycemic clamp. Therefore, our findings should be interpreted as comparisons with another surrogate marker rather than formal validation. Fourth, participants with prediabetes were excluded from the present analysis, as our primary objective was to evaluate the discriminatory ability between normoglycemia and T2DM; however, we acknowledge that prediabetes represents an important at-risk group, and future studies should extend these comparisons to this population. Fifth, the multivariate models were adjusted only for age; residual confounding by other metabolic or treatment-related factors (e.g., BMI, blood pressure, and medications) may therefore remain. Lastly, another limitation of our study is represented by the small number of subjects assigned to each subgroup in the logistic regression analysis, which may justify the wide confidence intervals, and by the fact that we evaluated a large number of IR indicators without applying a formal correction for multiple comparisons, which increases the risk of type I error and suggests that some associations may be spurious.

Our study also presents several strengths. One of these strengths is represented by the novelty of some IR indicators, comparing their predictive capacity in T2DM diagnosis. Moreover, the sample size, but also the method of its calculation and the wide age spectrum, ensure a high representativeness. In addition, the collection of anthropometric and biochemical data was carried out through standardized measurements. Another strength is represented by the fact that T2DM diagnosis was based on the evaluation of FPG but also of 2 h plasma glucose during OGTT and HbA1c, establishing the diagnosis through the recommendations of current guidelines.

The subject of our research has not been sufficiently investigated, and replication in multiethnic, multicenter cohorts is necessary to increase relevance and provide results validation. Longitudinal research is needed in order to establish the cause–effect relationship between IR biomarkers and T2DM. All this highlights the usefulness of identifying early biomarkers of T2DM, which is extremely important in countries with limited health systems due to the low cost but also because they can be easily calculated by considering routine biochemical data. IR indicators can thus facilitate clinical practice and can be useful in T2DM prevention.

## 4. Materials and Methods

### 4.1. Participants

The PREDATORR study was conducted in Romania between 2012 and 2014 as a cross-sectional, population-based study and aimed to determine the prevalence of DM, prediabetes, overweight and obesity, dyslipidemia, hyperuricemia, and chronic kidney disease (CKD) in the adult population, aged between 20 and 79 years. The design of the PREDATORR study has been previously described in articles published by colleagues from our team [4,29]. Participants were enrolled in the study by the computerized random selection method using the databases of 101 general practitioners who are found in the database of the National Health Insurance Company, who were also randomly enrolled. The selection was carried out equally, in relation to the eight historical regions of our country. In order for the sample to be representative of the adult population of Romania, based on the most recent census available at that time, namely the 2002 Romanian Census, a total of 2728 subjects were enrolled in the study. Inclusion criteria for the present study were represented by the age of participants being between 20 and 79 years, being born in Romania, those who had lived in the country for the last 10 years and are included on the list of a general practitioner, and those diagnosed with T2DM or normoglycemia. Participants with prediabetes (n = 580) were excluded from the present analysis, as the primary aim was to assess the discriminatory ability of insulin resistance indicators between normoglycemic and T2DM groups. Other exclusion criteria used in this analysis also included other forms of DM, as well as pregnant or breastfeeding women. Moreover, written informed consent was obtained from all study participants before undergoing any specific procedure. The PREDATORR study was conducted in accordance with the World Medical Association Declaration of Helsinki—Ethical Principles for Medical Research Involving Human Participants, as well as with the applicable standards of the International Conference on Harmonization (ICH)/Good Clinical Practice (GCP) and was approved by the Romanian National Ethics Committee (approval code 4064 from 12 December 2012).

### 4.2. Anamnestic, Socio-Demographic, and Lifestyle Data

A questionnaire was used to collect information regarding the lifestyle adopted by the participants (diet, smoking, alcohol consumption, and sleep duration), socio-demographic characteristics (gender, age, marital status, and education level), medical history of DM, and also the antidiabetic treatment used at the time of enrollment. Data on smoking were collected using a self-administered questionnaire; thus, participants were divided according to smoking status into the following categories: non-smokers (subjects had never smoked), current smokers (subjects smoked more than one cigarette per day, either daily or occasionally, and had not quit smoking), and former smokers (subjects who had quit smoking). Participants who denied drinking alcohol in the past month were classified as non-drinkers. Subjects who slept less than 7 h per night were considered to have a reduced sleep duration. Education level was classified as low if they had completed primary or secondary school and high if they had completed high school and college, or university.

### 4.3. Clinical and Biochemical Data

The recorded clinical data was represented by anthropometric data, such as height, weight, and also waist circumference (WC), hip circumference, and neck circumference (NC). All these measurements are non-invasive, cost-effective, and easy to use. Systolic blood pressure (SBP) and diastolic blood pressure (DBP) were also measured, using standard procedures. Following the recording of anthropometric data, we calculated the body mass index (BMI) using the following formula: BMI = weight (kilograms)/height squared (meters); the results were classified according to the World Health Organization (WHO) criteria [59], namely: subjects with BMI 25–29.9 kg/m^2^ were classified as overweight, and obesity was defined as BMI ≥ 30 kg/m^2^. Moreover, WC ≥ 80 cm in women and ≥94 cm in men was used as criteria to define abdominal obesity. We also calculated the waist-to-height ratio (WHtR) according to the formula WC (cm)/height (cm), abdominal obesity being characterized by a value ≥ 0.5. The neck-circumference-to-height ratio (NHtR) was also calculated according to the formula: NC (cm)/height (cm). HOMA-IR was estimated by applying the equation proposed by Matthews in 1985 [18].

Biochemical data was obtained by collecting blood samples under fasting conditions, and the analyses were performed according to standardized procedures. Enzymatic methods were used to determine fasting plasma glucose (FPG), total cholesterol (TC), high-density lipoprotein cholesterol (HDL-c), and low-density lipoprotein cholesterol (LDL-c), as well as triglycerides (TG). In addition, the technique used to determine the glycated hemoglobin (HbA1c) was represented by the immunoturbidimetric method. The diagnosis of DM was established according to the American Diabetes Association (ADA) guidelines [5], taking into account the values of FPG, HbA1c, 2 h plasma glucose during the oral glucose tolerance test (OGTT), and the presence of hyperglycemia symptoms, as well as self-reported diagnosis. OGTT was performed when the individual was fasting for at least 8 h, using 75 g of anhydrous glucose dissolved in 250–300 mL of water; blood glucose was measured fasted and at 2 h.

Confirmation of the diagnosis of DM, in the absence of unequivocal hyperglycemia, required two altered test results presented in Table 7, measured at the same time or at two different time points [5].

IR indices, calculated according to previously published formulas, are summarized in Table 8; the three CHG derivatives (CHG–WHtR, CHG–NC, and CHG–NHtR) are newly proposed in the present study.

### 4.4. Statistical Analysis

The study used a cluster sampling design to select the study participants. The Kolmogorov–Smirnov test was used in order to identify the distribution of continuous variables. Data that showed normal distribution were presented as mean ± standard deviation (SD), while data that had a non-Gaussian distribution were presented as median and interquartile range (IQR). In order to establish the significance of differences between groups, the Mann–Whitney U test was used to compare medians, and Student’s t-test was used to compare means. In addition, the chi-square test was used to analyze categorical variables. The IR biomarkers’ cut-off value was established by analyzing the area under the receiver operating characteristic (AUROC) curve. Univariate and multivariate logistic regressions aimed to identify the relationships between IR indicators and T2DM but also the risk factors of this disease, resulting in odds ratio (OR) with 95% confidence interval (CI). Multivariate logistic regression models were adjusted only for age. Additional adjustment for variables such as BMI, blood pressure, or lipid levels was not performed, as these parameters are inherently correlated with the IR indices under study and could introduce collinearity, potentially obscuring the associations of interest. HbA1c was modeled as a continuous variable, with ORs expressed per 1% increment. The analyses of this study were performed according to participants’ gender, and statistical significance was obtained if *p* value < 0.05 was recorded. Data analysis was performed using Statistical Package for the Social Sciences (SPSS) version 26.0 (SPSS Inc., Chicago, IL, USA).

## 5. Conclusions

In this study, TyG–WC, TyG–NC, TG/HDL-c, CHG–WC, and CHG–NC indicators had higher values in the male group, while TyG, TyG–BMI, TyG–WHtR, TyG–NHtR, MetS-IR, CHG, CHG–BMI, CHG–WHtR, and CHG–NHtR had similar values, regardless of gender. In addition, in both studied groups, all the analyzed indicators presented statistically significantly higher values in T2DM (+) participants compared to T2DM (−) (*p* < 0.001). Among all these biomarkers, regardless of subject’s gender, CHG–WHtR, an indicator developed by our team, had the largest AUROC curve, followed by CHG–WC and TyG–WHtR, thus demonstrating a strong discriminatory ability for T2DM compared to other indicators, including HOMA-IR. A ROC curve analysis revealed, both in males and females, similar AUROC curves to HOMA-IR for TyG, TyG–WC, TyG–WHtR, CHG, CHG–WC, and CHG–WHtR, with a comparable sensitivity and specificity, these being considered important results in daily clinical practice given the limited applicability of HOMA-IR. Also, regardless of participant’s gender, TyG and CHG were independent predictive factors for T2DM. By introducing three new CHG derivatives (CHG–WHtR, CHG–NC, and CHG–NHtR) and directly comparing them with HOMA-IR, our study provides original evidence on the potential utility of simple, non-invasive IR markers for identifying individuals at risk of T2DM in the general population. Our study results may provide new insights for diagnosis, as well as methodologies for the early identification of T2DM and implementation of individualized prevention strategies.

## Figures and Tables

**Table 1:** Demographic, anthropometric, and paraclinical characteristics of the study participants.

Characteristics	Male Group			Female Group		
	T2DM (+)	T2DM (−)	*p* Value	T2DM (+)	T2DM (−)	*p* Value
Participants, no.	214	777		183	906	
Age (years)	63 (12)	55 (23)	<0.001	64 (12)	54 (22)	<0.001
BMI (kg/m^2^)	30.6 (6.3)	27.1 (5.3)	<0.001	31.6 (7.1)	26.5 (7.9)	<0.001
WC (cm)	110 (15)	100 (15)	<0.001	104 (15)	90 (20)	<0.001
WHtR	0.62 (0.10)	0.57 (0.08)	<0.001	0.65 ± 0.07	0.56 ± 0.09	<0.001
NC (cm)	42 (4)	40 (4)	<0.001	37 (4)	35 (5)	<0.001
NHtR	0.24 (0.03)	0.22 (0.03)	<0.001	0.23 (0.03)	0.21 (0.03)	<0.001
SBP (mmHg)	147 (30)	136 (26)	<0.001	140 (31)	130 (27)	<0.001
DBP (mmHg)	81 (16)	80 (17)	0.137	80 (16)	79 (16)	0.168
FPG (mg/dL)	121.5 (47)	82.0 (17)	<0.001	123 (45)	80 (15)	<0.001
HbA1c (%)	6.73 (1.7)	5.30 (0.4)	<0.001	6.7 (1.2)	5.3 (0.4)	<0.001
TC (mg/dL)	185.5 (60)	202.0 (57)	<0.001	208 (72)	206 (62)	0.368
HDL-c (mg/dL)	44.0 (15)	48.6 (18)	<0.001	51.6 (18)	58.0 (19)	<0.001
LDL-c (mg/dL)	111.1 (50)	124.0 (51)	<0.001	124 (68)	127 (53)	0.196
TG (mg/dL)	136.5 (113)	119.0 (84)	<0.001	138.4 (74)	101.0 (67)	<0.001
HOMA-IR	3.83 (3.68)	1.68 (1.67)	<0.001	4.10 (3.53)	1.62 (1.36)	<0.001
Smoking (%)						
Daily smokers	12.9	24.3	<0.001	8.7	16.1	0.004
Occasional smokers	1.4	4.5		0.5	3.0	
Former smokers	47.9	37.4		23.4	16.4	
Non-smokers	37.8	33.8		67.4	64.5	
Alcohol consumption (%)	66.4	82.0	<0.001	29.9	36.0	0.115
Reduced sleep duration (%)	28.6	27.1	0.667	42.7	34.1	0.026
Marital status (%)						
Married	85.3	81.5	0.002	61.4	67.2	<0.001
Single	2.8	10.2		5.4	8.5	
Divorced	5.5	4.7		4.3	8.1	
Widowed	6.5	3.6		28.8	16.2	
High educational level (%)	57.9	61.4	0.354	50.8	67.5	<0.001

T2DM: type 2 diabetes mellitus; BMI: body mass index; WC: waist circumference; WHtR: waist-to-height ratio; NC: neck circumference; NHtR: neck-circumference-to-height ratio; SBP: systolic blood pressure; DBP: diastolic blood pressure; FPG: fasting plasma glucose; HbA1c: glycated hemoglobin; TC: total cholesterol; HDL-c: high-density lipoprotein cholesterol; LDL-c: low-density lipoprotein cholesterol; TG: triglycerides; and HOMA-IR: homeostatic model assessment for IR. Continuous variables with abnormal distribution are presented as median (IQR), and those with normal distribution are presented as mean ± standard deviation.

**Table 2:** IR biomarkers according to gender and T2DM.

IR Indicators	Male Group			Female Group		
	T2DM (+)	T2DM (−)	*p* Value	T2DM (+)	T2DM (−)	*p* Value
TyG	9.11 (0.95)	8.49 (0.76)	<0.001	9.05 (0.71)	8.30 (0.70)	<0.001
TyG–BMI	284.83 (73.39)	230.78 (53.87)	<0.001	286.16 (68.54)	223.17 (76.22)	<0.001
TyG–WC	999.36 (211.54)	853.76 (171.75)	<0.001	945.11 ± 138.12	759.66 ± 148.10	<0.001
TyG–WHtR	5.84 (1.27)	4.86 (0.97)	<0.001	5.92 (1.01)	4.68 (1.31)	<0.001
TyG–NC	388.24 (75.08)	343.33 (60.41)	<0.001	339.21 (59.49)	290.88 (55.38)	<0.001
TyG–NHtR	2.25 (0.45)	1.96 (0.34)	<0.001	2.11 (0.34)	1.79 (0.36)	<0.001
TG/HDL-c	3.45 (4.01)	2.43 (2.46)	<0.001	2.71 (2.22)	1.73 (1.57)	<0.001
MetS-IR	49.93 (14.09)	39.71 (11.13)	<0.001	48.12 (11.86)	36.93 (13.48)	<0.001
CHG	5.61 (0.69)	5.12 (0.52)	<0.001	5.46 (0.61)	4.94 (0.48)	<0.001
CHG–BMI	171.85 (47.13)	139.20 (34.07)	<0.001	174.05 (46.81)	134.11 (45.77)	<0.001
CHG–WC	611.01 (135.58)	512.32 (106.83)	<0.001	573.58 (110.58)	450.39 (128.17)	<0.001
CHG–WHtR	3.55 (0.88)	2.92 (0.59)	<0.001	3.62 (0.71)	2.79 (0.79)	<0.001
CHG–NC	236.70 (48.05)	206.29 (36.13)	<0.001	207.54 (38.01)	173.40 (34.90)	<0.001
CHG–NHtR	1.37 (0.26)	1.17 (0.20)	<0.001	1.29 (0.21)	1.07 (0.22)	<0.001

IR: insulin resistance; T2DM: type 2 diabetes mellitus; TyG: triglyceride–glucose; TyG–BMI: TyG–body mass index; TyG–WC: TyG–waist circumference; TyG–WHtR: TyG–waist-to-height ratio; TyG–NC: TyG–neck circumference; TyG–NHtR: TyG–neck-circumference-to-height ratio; TG/HDL-c: triglyceride to high-density-lipoprotein cholesterol; MetS-IR: metabolic score for insulin resistance; CHG: cholesterol, HDL, and glucose; CHG–BMI: CHG–body mass index; CHG–WC: CHG–waist circumference; CHG–WHtR: CHG–waist-to-height ratio; CHG–NC: CHG–neck circumference; and CHG–NHtR: CHG–neck-circumference-to-height ratio. Continuous variables with abnormal distribution are presented as median (IQR), and those with normal distribution are presented as mean ± standard deviation.

**Table 3:** The ROC curve analysis for the IR biomarkers in males.

IR Indicators	AUROC Curve	Standard Error	95% CI	*p* Value	Cut-Off Value	Sensitivity (%)	Specificity (%)
CHG–WHtR	0.809	0.017	0.775–0.842	<0.001	3.22	70.7	75.3
CHG–WC	0.799	0.017	0.765–0.833	<0.001	560.59	69.8	73.8
TyG–WHtR	0.791	0.018	0.756–0.826	<0.001	5.37	69.3	75.8
CHG	0.784	0.019	0.746–0.821	<0.001	5.32	72.2	71.7
TyG–WC	0.782	0.018	0.747–0.818	<0.001	932.31	69.3	74.3
HOMA-IR	0.779	0.019	0.742–0.816	<0.001	2.63	71.6	74.4
CHG–BMI	0.776	0.018	0.740–0.812	<0.001	158.77	63.7	77.9
CHG–NHtR	0.771	0.019	0.734–0.809	<0.001	1.25	73.5	70.0
TyG–BMI	0.762	0.019	0.725–0.799	<0.001	261.03	64.2	76.0
MetS-IR	0.762	0.019	0.725–0.799	<0.001	44.60	67.4	70.4
CHG–NC	0.760	0.019	0.722–0.798	<0.001	223.84	66.0	76.2
TyG	0.751	0.020	0.713–0.790	<0.001	8.92	65.7	76.1
TyG–NHtR	0.744	0.020	0.705–0.783	<0.001	2.07	71.6	69.0
TyG–NC	0.735	0.020	0.696–0.774	<0.001	368.23	65.1	72.0
TG/HDL-c	0.629	0.022	0.587–0.672	<0.001	2.52	68.1	52.5

IR: insulin resistance; ROC: receiver operating characteristic; AUROC: area under the receiver operating characteristic; CI: confidence interval; TyG: triglyceride–glucose; TyG–BMI: TyG–body mass index; TyG–WC: TyG–waist circumference; TyG–WHtR: TyG–waist-to-height ratio; TyG–NC: TyG–neck circumference; TyG–NHtR: TyG–neck-circumference-to-height ratio; TG/HDL-c: triglyceride to high-density-lipoprotein cholesterol; MetS-IR: metabolic score for insulin resistance; CHG: cholesterol, HDL, and glucose; CHG–BMI: CHG–body mass index; CHG–WC: CHG–waist circumference; CHG–WHtR: CHG–waist-to-height ratio; CHG–NC: CHG–neck circumference; CHG–NHtR: CHG–neck-circumference-to-height ratio; and HOMA-IR: homeostasis model assessment of insulin resistance. Indicators are presented in descending order of AUROC values.

**Table 4:** The ROC curve analysis for the IR biomarkers in females.

IR Indicators	AUROC Curve	Standard Error	95% CI	*p* Value	Cut-Off Value	Sensitivity (%)	Specificity (%)
CHG–WHtR	0.840	0.015	0.811–0.869	<0.001	3.20	79.3	75.5
TyG	0.837	0.016	0.805–0.868	<0.001	8.70	77.7	76.9
HOMA-IR	0.837	0.016	0.805–0.870	<0.001	2.52	79.5	76.9
TyG–WHtR	0.834	0.015	0.805–0.864	<0.001	5.33	78.8	75.2
CHG–WC	0.826	0.016	0.794–0.857	<0.001	510.35	77.7	74.2
TyG–WC	0.821	0.016	0.790–0.853	<0.001	854.88	76.6	74.7
CHG–NHtR	0.818	0.017	0.786–0.851	<0.001	1.17	77.3	74.4
CHG	0.813	0.018	0.777–0.848	<0.001	5.19	76.8	73.8
TyG–NHtR	0.813	0.016	0.781–0.845	<0.001	1.94	79.3	70.6
CHG–BMI	0.803	0.016	0.772–0.835	<0.001	159.60	70.3	76.4
CHG–NC	0.797	0.018	0.761–0.833	<0.001	193.66	68.6	79.2
TyG–BMI	0.793	0.016	0.762–0.825	<0.001	245.02	83.2	64.9
TyG–NC	0.791	0.018	0.756–0.826	<0.001	315.03	74.6	73.2
MetS-IR	0.787	0.017	0.754–0.819	<0.001	42.72	76.2	70.3
TG/HDL-c	0.691	0.021	0.650–0.731	<0.001	2.20	65.9	64.6

IR: insulin resistance; ROC: receiver operating characteristic; AUROC: area under the receiver operating characteristic; CI: confidence interval; TyG: triglyceride–glucose; TyG–BMI: TyG–body mass index; TyG–WC: TyG–waist circumference; TyG–WHtR: TyG–waist-to-height ratio; TyG–NC: TyG–neck circumference; TyG–NHtR: TyG–neck-circumference-to-height ratio; TG/HDL-c: triglyceride to high-density-lipoprotein cholesterol; MetS-IR: metabolic score for insulin resistance; CHG: cholesterol, HDL, and glucose; CHG–BMI: CHG–body mass index; CHG–WC: CHG–waist circumference; CHG–WHtR: CHG–waist-to-height ratio; CHG–NC: CHG–neck circumference; CHG–NHtR: CHG–neck-circumference-to-height ratio; and HOMA-IR: homeostasis model assessment of insulin resistance. Indicators are presented in descending order of AUROC values.

**Table 5:** Variables associated with T2DM from univariate logistic regression analysis.

Variables	Male Group			Female Group		
	OR	95% CI	*p* Value	OR	95% CI	*p* Value
Age (years)	1.058	1.044–1.072	<0.001	1.073	1.057–1.090	<0.001
BMI (kg/m^2^)	1.181	1.139–1.224	<0.001	1.146	1.114–1.179	<0.001
WC (cm)	1.067	1.052–1.081	<0.001	1.068	1.054–1.082	<0.001
WHtR (≥0.5995)	4.641	3.366–6.400	<0.001	5.439	3.816–7.752	<0.001
NC (cm)	1.050	1.024–1.078	<0.001	1.136	1.092–1.182	<0.001
NHtR (≥0.2305)	2.908	2.092–4.040	<0.001	3.591	2.593–4.972	<0.001
SBP (mmHg)	1.021	1.014–1.029	<0.001	1.023	1.016–1.030	<0.001
DBP (mmHg)	1.010	0.997–1.022	0.122	1.009	0.996–1.023	0.176
FPG (mg/dL)	1.073	1.061–1.084	<0.001	1.097	1.083–1.112	<0.001
HbA1c (%) *	40.877	23.731–70.412	<0.001	3.968	3.135–5.022	<0.001
TC (mg/dL)	0.994	0.990–0.997	<0.001	0.998	0.995–1.001	0.224
HDL-c (mg/dL)	0.966	0.954–0.978	<0.001	0.968	0.957–0.980	<0.001
LDL-c (mg/dL)	0.990	0.986–0.994	<0.001	0.997	0.993–1.001	0.127
TG (mg/dL)	1.003	1.002–1.004	<0.001	1.005	1.003–1.007	<0.001
HOMA-IR	1.289	1.213–1.370	<0.001	1.391	1.296–1.494	<0.001
TyG	5.849	4.222–8.103	<0.001	11.543	7.890–16.885	<0.001
TyG–BMI	5.662	4.092–7.835	<0.001	9.182	6.095–13.834	<0.001
TyG–WC	6.540	4.690–9.121	<0.001	9.705	6.679–14.102	<0.001
TyG–WHtR	7.018	5.025–9.801	<0.001	11.137	7.579–16.366	<0.001
TyG–NC	4.799	3.477–6.623	<0.001	8.003	5.567–11.505	<0.001
TyG–NHtR	5.366	3.840–7.498	<0.001	9.179	6.229–13.527	<0.001
TG/HDL-c	2.342	1.701–3.223	<0.001	3.442	2.466–4.805	<0.001
MetS-IR	4.929	3.561–6.823	<0.001	7.596	5.257–10.974	<0.001
CHG	6.233	4.454–8.723	<0.001	9.204	6.343–13.356	<0.001
CHG–BMI	6.200	4.473–8.594	<0.001	7.654	5.387–10.874	<0.001
CHG–WC	6.506	4.663–9.076	<0.001	10.008	6.856–14.610	<0.001
CHG–WHtR	7.289	5.206–10.206	<0.001	11.844	8.033–17.462	<0.001
CHG–NC	6.233	4.491–8.652	<0.001	8.344	5.879–11.844	<0.001
CHG–NHtR	6.166	4.391–8.660	<0.001	9.911	6.759–14.533	<0.001

OR: odds ratio; CI: confidence interval; BMI: body mass index; WC: waist circumference; WHtR: waist-to-height ratio; NC: neck circumference; NHtR: neck-circumference-to-height ratio; SBP: systolic blood pressure; DBP: diastolic blood pressure; FPG: fasting plasma glucose; HbA1c: glycated hemoglobin; TC: total cholesterol; HDL-c: high-density lipoprotein cholesterol; LDL-c: low-density lipoprotein cholesterol; TG: triglycerides; HOMA-IR: homeostatic model assessment for IR; TyG: triglyceride–glucose; TyG–BMI: TyG–body mass index; TyG–WC: TyG–waist circumference; TyG–WHtR: TyG–waist-to-height ratio; TyG–NC: TyG–neck circumference; TyG–NHtR: TyG–neck-circumference-to-height ratio; TG/HDL-c: triglyceride to high-density-lipoprotein cholesterol; MetS-IR: metabolic score for insulin resistance; CHG: cholesterol, HDL, and glucose; CHG–BMI: CHG–body mass index; CHG–WC: CHG–waist circumference; CHG–WHtR: CHG–waist-to-height ratio; CHG–NC: CHG–neck circumference; and CHG–NHtR: CHG–neck-circumference-to-height ratio. * ORs for HbA1c are expressed per 1% increase. The difference between males and females reflects different effect sizes and the smaller number of female cases, which may increase variability of the estimates.

**Table 6:** The multivariate logistic regression analysis model adjusted for age.

Variables	Male Group			Female Group		
	OR	95% CI	*p* Value	OR	95% CI	*p* Value
TyG	2.994	1.656–5.412	<0.001	8.170	3.779–17.665	<0.001
TyG–BMI	1.303	0.564–3.010	0.535	1.987	0.869–4.542	0.104
TyG–WC	2.221	1.031–4.787	0.042	0.818	0.318–2.099	0.676
TyG–WHtR	1.243	0.582–2.655	0.574	1.843	0.764–4.449	0.174
TyG–NC	0.529	0.234–1.198	0.127	1.085	0.480–2.449	0.845
TyG–NHtR	1.191	0.534–2.658	0.669	0.864	0.382–1.950	0.724
TG/HDL-c	0.371	0.201–0.683	0.001	0.176	0.085–0.364	<0.001
MetS-IR	1.018	0.504–2.053	0.961	0.977	0.390–2.446	0.960
CHG	2.927	1.681–5.096	<0.001	2.004	1.105–3.636	0.022
CHG–BMI	1.798	0.832–3.887	0.136	1.429	0.669–3.052	0.356
CHG–WC	0.617	0.284–1.339	0.222	0.818	0.318–2.107	0.678
CHG–WHtR	1.030	0.492–2.156	0.936	1.699	0.670–4.308	0.264
CHG–NC	2.734	1.334–5.601	0.006	1.675	0.779–3.599	0.186
CHG–NHtR	1.054	0.507–2.190	0.888	1.319	0.586–2.969	0.503

OR: odds ratio; CI: confidence interval; TyG: triglyceride–glucose; TyG–BMI: TyG–body mass index; TyG–WC: TyG–waist circumference; TyG–WHtR: TyG–waist-to-height ratio; TyG–NC: TyG–neck circumference; TyG–NHtR: TyG–neck-circumference-to-height ratio; TG/HDL-c: triglyceride to high-density-lipoprotein cholesterol; MetS-IR: metabolic score for insulin resistance; CHG: cholesterol, HDL, and glucose; CHG–BMI: CHG–body mass index; CHG–WC: CHG–waist circumference; CHG–WHtR: CHG–waist-to-height ratio; CHG–NC: CHG–neck circumference; and CHG–NHtR: CHG–neck-circumference-to-height ratio.

**Table 7:** Criteria for diagnosis of DM (adapted from [5]).

Measure	Categorical Cut Points
FPG	≥126 mg/dL (7.0 mmol/L)
or	
2 h plasma glucose during OGTT	≥200 mg/dL (11.1 mmol/L)
or	
HbA1c	≥6.5% (48 mmol/mol)
or	
A random plasma glucose in an individual with classic symptoms of hyperglycemia or hyperglycemic crisis *	≥200 mg/dL (11.1 mmol/L)

* Classic hyperglycemic symptoms: polydipsia, polyuria, and unexplained weight loss. Hyperglycemic crisis refers to diabetic ketoacidosis and/or hyperglycemic hyperosmolar state.

**Table 8:** Overview of insulin resistance biomarkers.

Biomarker	Formula	Reference
HOMA-IR	(Fasting Insulin [µU/mL] × Fasting Glucose [mmol/L])/22.5	[18]
TyG index	Ln [Fasting Triglycerides (mg/dL) × Fasting Glucose (mg/dL)/2]	[20,60]
TyG–BMI	TyG × BMI (kg/m^2^)	[61]
TyG–WC	TyG × WC	[61]
TyG–WHtR	TyG × WHtR	[62]
TyG–NC	TyG × NC	[30]
TyG–NHtR	TyG × NHtR	[30]
TG/HDL-c	Triglycerides (mg/dL)/HDL-C (mg/dL)	[63]
METS-IR	(Ln [2 × Fasting Glucose (mg/dL) + Fasting Triglycerides (mg/dL)]) × BMI/(Ln [HDL-C (mg/dL)])	[53]
CHG index	Ln [Total Cholesterol (mg/dL) × Fasting Glucose (mg/dL)/HDL-C (mg/dL)]	[12]
CHG–BMI	CHG × BMI	[12]
CHG–WC	CHG × WC	[12]
CHG–WHtR	CHG × WHtR	Present study
CHG–NC	CHG × NC	Present study
CHG–NHtR	CHG × NHtR	Present study

TyG: triglyceride–glucose; TyG–BMI: TyG–body mass index; TyG–WC: TyG–waist circumference; TyG–WHtR: TyG–waist-to-height ratio; TyG–NC: TyG–neck circumference; TyG–NHtR: TyG–neck-circumference-to-height ratio; TG/HDL-c: triglyceride to high-density-lipoprotein cholesterol; MetS-IR: metabolic score for insulin resistance; CHG: cholesterol, HDL, and glucose; CHG–BMI: CHG–body mass index; CHG–WC: CHG–waist circumference; CHG–WHtR: CHG–waist-to-height ratio; CHG–NC: CHG–neck circumference; and CHG–NHtR: CHG–neck-circumference-to-height ratio. Ln represents natural logarithm.

## Data Availability

All data are contained in the manuscript.

## Abbreviations

The following abbreviations are used in this manuscript:

ADA	American Diabetes Association
AUROC	area under the receiver operating characteristic
BMI	body mass index
CHG	cholesterol, HDL, glucose
CHG–BMI	CHG–body mass index
CHG–NC	CHG–neck circumference
CHG–NHtR	CHG–neck-circumference-to-height ratio
CHG–WC	CHG–waist circumference
CHG–WHtR	CHG–waist-to-height ratio
CI	confidence interval
CKD	chronic kidney disease
DBP	diastolic blood pressure
DM	diabetes mellitus
FPG	fasting plasma glucose
FFA	free fatty acids
GCP	good clinical practice
HbA1c	glycated hemoglobin
HDL-c	high-density lipoprotein cholesterol
HOMA-IR	homeostasis model assessment of insulin resistance
ICH	International Conference on Harmonization
IDF	International Diabetes Federation
IQR	interquartile range
IR	insulin resistance
LDL-c	low-density lipoprotein cholesterol
MASLD	metabolic dysfunction-associated steatotic liver disease
MetS	metabolic syndrome
METS-IR	metabolic score for insulin resistance
NC	neck circumference
NHtR	neck-circumference-to-height ratio
OGTT	oral glucose tolerance test
OR	odds ratio
PREDATORR	PREvalence of DiAbeTes mellitus, pre diabetes, overweight, Obesity, dyslipidemia, hyperuricemia and chronic kidney disease in Romania
ROC	receiver operating characteristic
SBP	systolic blood pressure
SD	standard deviation
SPSS	statistical package for the social sciences
T2DM	type 2 diabetes mellitus
TC	total cholesterol
TG	triglycerides
TG/HDL-c	triglyceride to high-density-lipoprotein cholesterol
TyG	triglyceride-glucose
TyG–BMI	TyG–body mass index
TyG–NC	TyG–neck circumference
TyG–NHtR	TyG–neck-circumference-to-height ratio
TyG–WC	TyG–waist circumference
TyG–WHtR	TyG–waist-to-height ratio
WC	waist circumference
WHO	World Health Organization
WHtR	waist-to-height ratio
